# High mucin 5AC expression predicts adverse postoperative recurrence and survival of patients with clear-cell renal cell carcinoma

**DOI:** 10.18632/oncotarget.15894

**Published:** 2017-03-04

**Authors:** Haijian Zhang, Yidong Liu, Huyang Xie, Weisi Liu, Qiang Fu, Dengfu Yao, Jiejie Xu, Jianxin Gu

**Affiliations:** ^1^ Department of Biochemistry and Molecular Biology, School of Basic Medical Sciences, Fudan University, Shanghai, China; ^2^ Research Center of Clinical Medicine, Affiliated Hospital of Nantong University, Jiangsu, China; ^3^ Department of Urology, Fudan University Shanghai Cancer Center, Shanghai, China

**Keywords:** mucin 5AC, clear-cell renal cell carcinoma, overall survival, recurrence-free survival, prognostic biomarker

## Abstract

**Background:**

Mucin 5AC (MUC5AC), as a member of secreted/gel-forming mucin family, was frequently found to be abnormally expressed in inflammation or malignant diseases. However, the clinic pathologic features and prognostic values of MUC5AC in clear-cell renal cell carcinoma (ccRCC) have not been reported up to now.

**Methods:**

MUC5AC expression was analyzed by immunohistochemistry on tissue microarrays. Kaplan-Meier survival curves, Univariate and Multivariate Cox analysis and newly-established nomogram model were performed to evaluate the prognostic value.

**Results:**

MUC5AC expression was firstly found to be up-regulated in patients with ccRCC, positively associated with tumor size, pN stage, lymphovascular invasion, Fuhrman grade, rahbdoid differentiation, sarcomatoid features, tumor necrosis, ECOG-PS and recurrence. Furthermore, MUC5AC expression might be contributed to risk stratification of ccRCC patients with TNM stage III+IV or Fuhrman grade 3 or 4 for overall survival (OS) and recurrence-free survival (RFS) analysis, and it was demonstrated to be negatively correlated with OS and RFS of ccRCC patients. What's more, MUC5AC was identified as a potential independent adverse prognostic factor; prediction accuracy of MUC5AC-based new nomogram model was drastically improved for OS and RFS of ccRCC patients.

**Conclusion:**

MUC5AC is a promising independent adverse prognostic factor for ccRCC patients, it maybe conducive to postoperative risk stratification and guide treatment in the future.

## INTRODUCTION

Renal cell carcinoma (RCC), as the eighth most common cancer in the world, accounts for 2%-3% of all adult malignancies [1]. Nearly 50% of these patients will eventually develop metastatic disease, 20-30% of patients may develop postoperative recurrence [2]. Approximately 80% of all diagnosed RCC cases was constituted by clear-cell renal cell carcinoma (ccRCC), the main subtype of RCC [3]. Accumulating evidence suggests that the alteration of many kinds of biomarkers and corresponding downstream pathways are involved in the initiation and progression of tumor. The disease-free survival (DFS) and overall survival (OS) have been distinctly improved by partial or whole nephrectomy for those patients diagnosed with early stage ccRCC [4]. However, the prognosis is markedly worse for those patients diagnosed with advanced or metastatic ccRCC [5]. In fact, the median survival periods for stage III and stage IV patients are dropped to approximately 3.1 years and 1.1 years, respectively [6]. Therefore, it is urgent to develop new molecular markers for early diagnosis and treatment, and find potential prognostic biomarkers to monitor progression or recurrence for ccRCC patients, and also required to find high-efficiency molecular therapeutic targets to improve outcomes for advanced ccRCC patients.

Mucus, as the slimy and viscoelastic secretion, is found to cover the epithelial surface of tubular organs, such as tracheobronchial, reproductive tracts, gastrointestinal, as well as other specialized organs [7]. Mucins, in charge of the biochemical and biophysical properties of mucus, are high molecular weight glycoproteins, and exert an influence on the renewal and differentiation of epithelial cell, cell adhesions, immune response, signal transduction and maintenance of homeostasis [8]. Mucins are largely composed of oligosaccharides and serine/threonine/proline residues (Ser-Thr-Pro) linked via O-glycosidic linkages. Mucins can be classified as 3 soluble mucins (MUC7, MUC8 and MUC9), 5 secreted/gel-forming mucins (MUC2, MUC5AC, MUC5B, MUC6 and MUC19), and 12 membrane combined mucins (MUC1, MUC3A, MUC3B, MUC4, MUC11, MUC12, MUC13, MUC15, MUC16, MUC17, MUC20 and MUC21) [9].

Mucin 5AC (MUC5AC), as a member of secreted/gel-forming mucin family, was primitively isolated from a human tracheobronchial cDNA library. MUC5AC contains tandem repeats of eight amino acids (TTSTTSAP), and can be mapped to a mucin cluster on chromosome 11p15 [10]. MUC5AC is found to be highly expressed in conjunctiva and lacrimal gland tissues (but not in cornea), goblet cells of the respiratory epithelium, superficial gastric epithelium, endocervix, pancreas and gallbladder. Under pathological conditions, MUC5AC expression has been reported to be altered in a variety of diseases [11]. Increasing studies have suggested that abnormal expression of MUC5AC is associated with inflammation or the development and progression of malignant diseases, which may be induced by the activation of signaling pathways in response to several factors, for example growth factors, inflammatory cytokines, and bacterial products [9, 12]. MUC5AC was reported to be up-regulated in a case of oncocytic papillary RCC (OPRCC) [10], although there is no data about the expression of MUC5AC or MUC5AC mRNA in RCC in TCGA database. Certainly, there is no report about clinical pathological features and prognosis value of MUC5AC in RCC up to now.

In this research, we evaluated the clinical pathological features of MUC5AC in ccRCC by immunohistochemical staining and explored its relation with clinical outcomes. Furthermore, risk stratification of ccRCC patients with different stages was analyzed. Finally, a nomogram integrating MUC5AC with other independent prognostic parameters was established to help monitor the prognosis and guide management of postoperative ccRCC patients.

## MATERIALS AND METHODS

### Patients and specimens

A total of 602 ccRCC patients treated with radical nephrectomy or nephron-sparing surgery Zhongshan Hospital in 2008 and 2009 were retrospectively recruited. Patients were included if they had histopathologically proven as ccRCC after radical or partial nephrectomy with complete medical records, and without the history of other malignancies or previous anticancer therapy. Meanwhile, patients were excluded if they had mixed type of primary kidney cancer via histopathological confirmation, tumors with necrosis > 80%, or died within the first month after surgery due to surgical complications. The ccRCC patients with N1 or M1 tumors at the time of surgery were excluded from recurrence-free survival (RFS) analysis. Patients were staged according to postoperative pathological data and radiographic reports [13].

Overall survival (OS) was defined as the period from the date of surgery to the date of death (or the last follow-up), and RFS was defined as the period from the date of surgery to the date of recurrence (or the last follow-up). Especially to deserve to be mentioned, postoperative patients with local recurrence and/or distal metastasis was regarded as the study endpoint of recurrence. The time of follow-up visit ranged from 39 to 74 months, averagely 67 months. Among the overall cohort of 602 patients, 102 (16.94%) patients died of all causes, while 108 (17.94%) patients had recurrence during the follow-up period. Written informed consent was obtained from each patient, and this research was approved by the Clinical Research Ethics Committee of Zhongshan Hospital, Fudan University (Shanghai, China).

### Tissue microarray, immunohistochemistry and evaluation of immunohistochemical MUC5AC expression

Tissue microarray was constructed by Formalin-fixed, paraffin embedded surgical specimens, and subsequently used for immunohistochemistry (IHC) study as previously described [14]. The MUC5AC expression was analyzed by immunohistochemistry staining with anti - MUC5AC antibody (1:100 dilution; Abcam, USA). Meanwhile, negative controls were synchronously carried out but without the primary antibody. As previously described, the staining intensity of immunohistochemical MUC5AC expression was scored independently by two ccRCC pathologists according to the semi-quantitative immunoreactivity scoring (IRS) system [15]. A semi-quantitative H-score (range: 0 to 300) was derived according to multiplying the staining intensity (0: negative, 1: weak, 2: moderate, 3: strong) and the percentage of immunoreactivity cells (0-100%) at each expression level for each sample. According to the “minimum *P* value” approach conducted by X-tile, as well as data distribution and mean, 141 was determined as the cutoff of IRS system, and the patients were subsequently dichotomized into low and high groups according to the optimum cutoff.

### Statistical analysis

Statistical analyses were performed with MedCalc Software 11.4.2.0 (Mariakerke, Belgium), X-tile software 3.6.1 (Yale University, New Haven, CT, USA), and R software 3.0.2 (R Foundation for Statistical Computing, Vienna, Austria). The optimum cutoff of immunohistochemical MUC5AC expression was selected according to “optimal p value method” with X-tile. The correlation between clinicopathological parameters of MUC5AC expression in ccRCC patients was analyzed by Student's t-test, χ^2^ test or Fisher's exact test. OS and RFS curve were drawn with the Kaplan–Meier method and their differences between groups were analyzed by the log-rank test. Nomogram and calibration plot were analyzed by R software with the “rms” package. All significance tests were two-sided and *P* < 0.05 was considered statistically significant.

## RESULTS

### Clinicopathological characteristics of immunohistochemical MUC5AC expression and its association with OS and RFS of the patients with ccRCC

**Figure 1:**
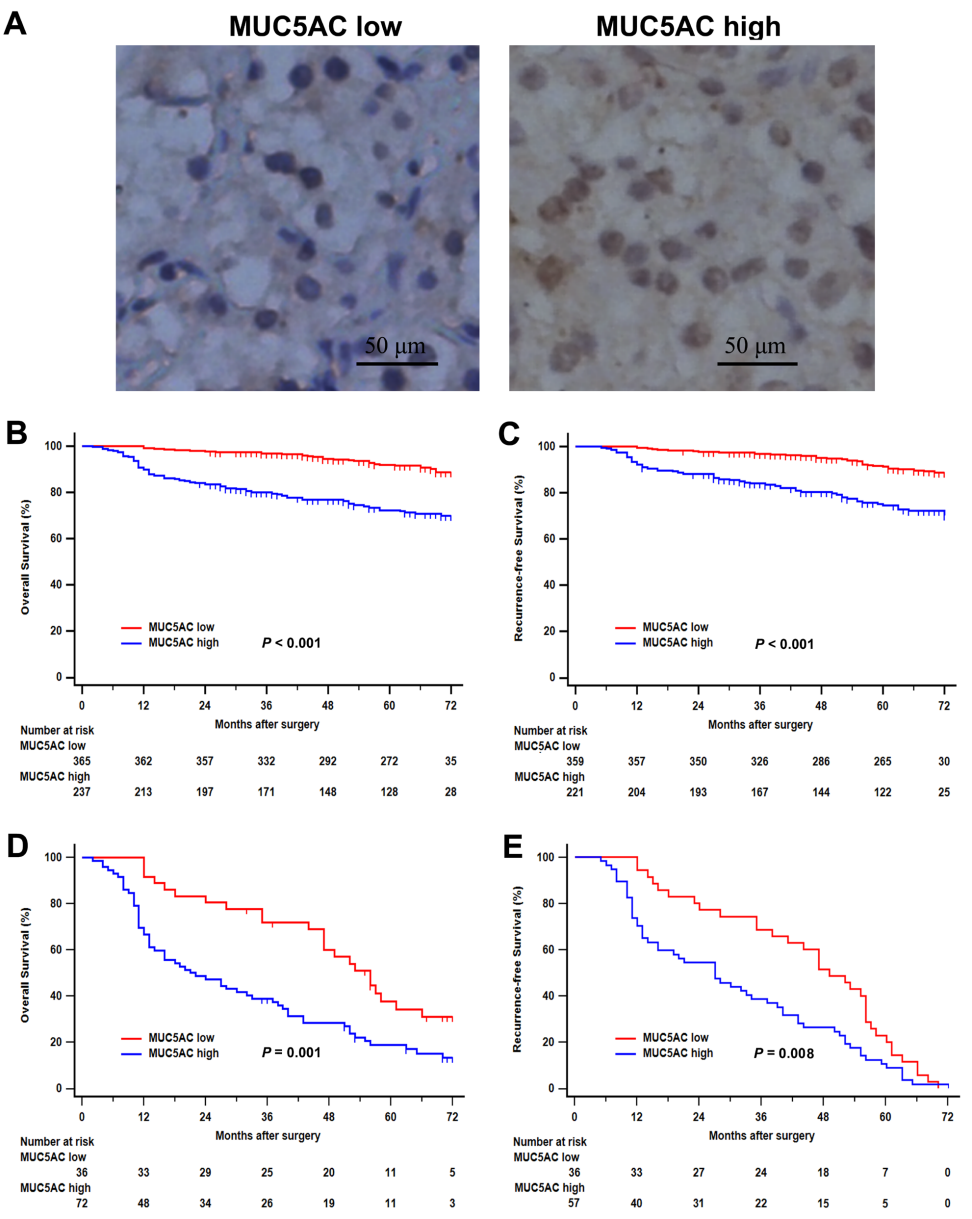
Immunohistochemical MUC5AC expression and its association with clinical outcomes of patients with clear-cell renal cell carcinoma (ccRCC) **A**., representative photographs of MUC5AC immunostaining. Low MUC5AC expression (left panel), high MUC5AC expression (right panel). Original magnification: ×200. **B**., overall survival analysis according to MUC5AC expression. **C**., recurrence-free survival analysis according to MUC5AC expression. **D**., overall survival analysis of patients with recurrence according to MUC5AC expression. **E**., recurrence-free survival analysis of patients with recurrence according to MUC5AC expression. *P* value was analyzed by log-rank test.

To investigate the clinic pathological characteristics of MUC5AC expression during the occurrence and progression of ccRCC, MUC5AC expression was analyzed by IHC staining in clinical specimens of 602 ccRCC patients. As shown in Figure 1A, the positive staining of MUC5AC was mainly located in cytoplasm and cell nucleus of tumor cells in ccRCC specimens. According to the optimum cutoff value, 39.37% (237/602) specimens were defined as high MUC5AC expression group, and the others were regarded as low MUC5AC expression group.

The correlation between MUC5AC expression and clinic-pathological features was shown in Table 1. Expression level of MUC5AC was positively correlated with pN stage (*P* = 0.001), tumor size (*P* < 0.001), Fuhrman grade (*P* < 0.001), lymphovascular invasion (LVI; *P* = 0.002), sarcomatoid features (*P* < 0.001), rahbdoid differentiation (*P* = 0.001), ECOG-PS (Eastern Cooperative Oncology Group performance status, *P* < 0.001), tumor necrosis (*P* < 0.001) and recurrence (*P* < 0.001). At the same time, no significant correlation was found between MUC5AC expression and other clinic pathological characteristics of ccRCC patients, such as age of patients, gender, pT – stage, metastasis.

**Table 1:** Correlation between MUC5AC expression and clinical characteristics in ccRCC patients

Characteristic	Patients		MUC5AC expression		
	No.	%	Low	High	*P*
**Mean age, years†**					**0.702**
**Mean ± SD**	55.00 ± 12.32		55.05 ± 12.22	55.33 ±12.50	
**Gender**					**0.804**
**Male**	422	70.10%	254	168	
**Female**	180	29.90%	111	69	
**Tumor size, cm†**					**< 0.001**
**Mean ± SD**	4.00 ± 2.55		4.01 ± 2.24	5.04 ± 2.85	
**pT stage**					**0.299**
**T1**	402	66.78%	250	152	
**T2**	48	7.97%	24	24	
**T3**	143	23.75%	87	56	
**T4**	9	1.50%	4	5	
**pN stage**					**0.001**
**N0**	591	98.17%	364	227	
**N1**	11	1.83%	1	10	
**Metastasis**					**0.289**
**M0**	590	98.01%	360	230	
**M1**	12	1.99%	5	7	
**Fuhrman grade**					**< 0.001**
**1**	93	15.45%	64	29	
**2**	244	40.53%	168	76	
**3**	158	26.25%	96	62	
**4**	107	17.77%	37	70	
**LVI**					**0.002**
**Absent**	442	73.42%	285	157	
**Present**	160	26.58%	80	80	
**Rahbdoid differentiation**					**0.001**
**Absent**	566	94.02%	353	213	
**Present**	36	5.98%	12	24	
**Sarcomatoid features**					**< 0.001**
**Absent**	576	95.68%	359	217	
**Present**	26	4.32%	6	20	
**Tumor necrosis**					**< 0.001**
**Absent**	453	75.25%	305	148	
**Present**	149	24.75%	60	89	
**ECOG-PS**					**< 0.001**
**0**	492	81.73%	317	175	
**≥1**	110	18.27%	48	62	
**Recurrence**					**< 0.001**
**Absent**	494	82.06%	329	165	
**Present**	108	17.94%	36	72	

MUC5AC, mucin 5AC; ccRCC, clear-cell renal cell carcinoma; LVI, lymphovascular invasion; ECOG-PS, Eastern Cooperative Oncology Group performance status. *P < 0.05 is considered statistically significant. †The results of continuous variables are presented as mean ± SD (standard deviation).

The association between MUC5AC expression and prognosis of patients with ccRCC was analyzed by Kaplan-Meier method and log-rank test. As shown in Figure 1B, patients with high MUC5AC expression level presented a poorer OS comparing with those with a low MUC5AC expression (*P* < 0.001). Similarly, patients with high MUC5AC expression level were confirmed to have worse outcomes for RFS comparing with those with a low MUC5AC expression (*P* < 0.001; Figure 1C). Considering as dataset “Recurrence” (108 patients), patients with high MUC5AC expression level were found to have a more adverse OS (*P* = 0.001; Figure 1D) and RFS (*P* = 0.008; Figure 1E), comparing with those with a low MUC5AC expression. Therefore, It was an undisputable fact that patients with higher MUC5AC expression level might have earlier death and recurrence.

### Risk stratification of ccRCC patients with different TNM stages or Fuhrman grades according to MUC5AC expression

**Figure 2:**
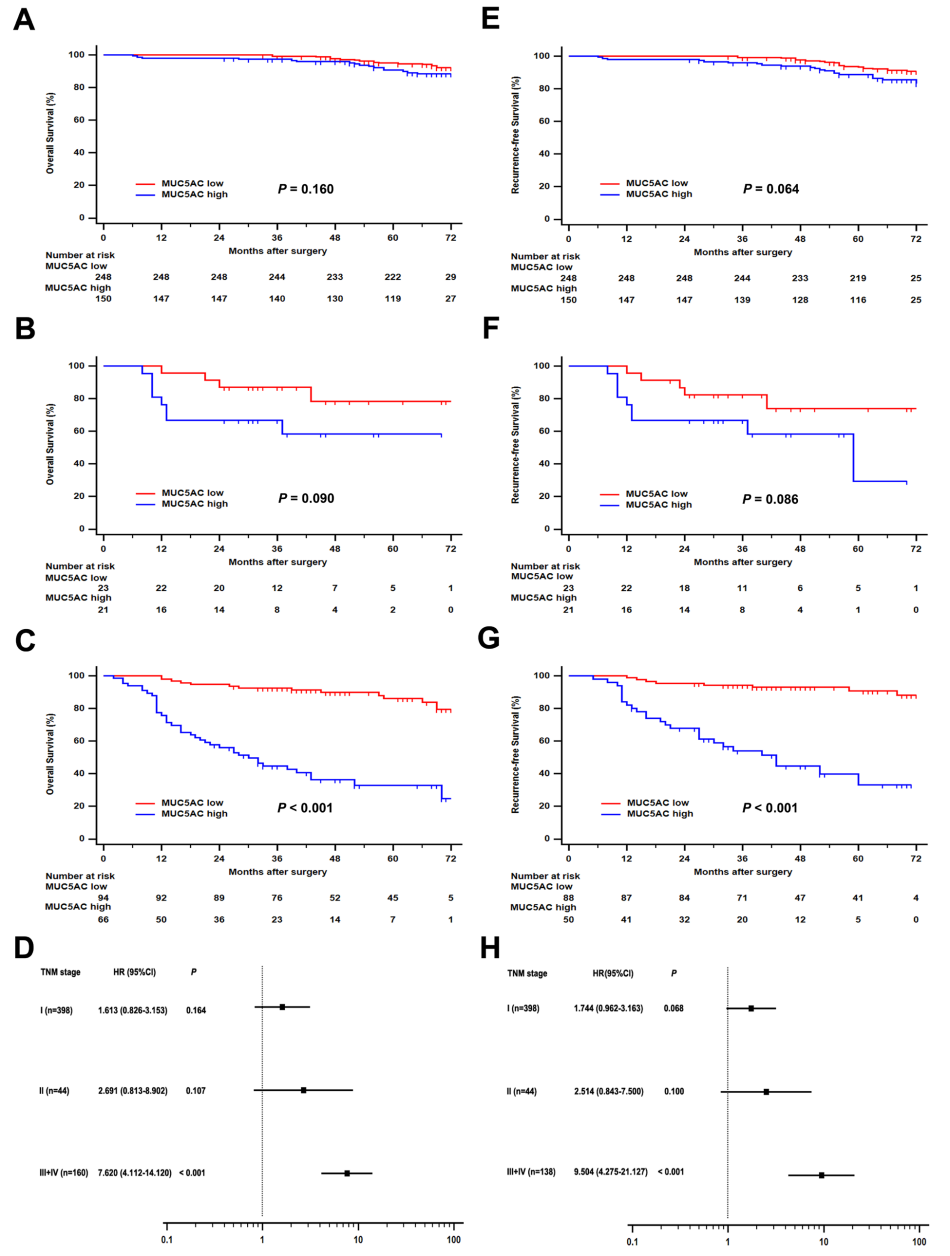
Subgroup analysis of MUC5AC prognostic value in ccRCC patients with different pT stages Overall survival (OS) for ccRCC patients with **A**. pT1 stage, or **B**. pT2 stage, or **C**. pT3+4 stages. Recurrence-free survival (RFS) for ccRCC patients with **E**. pT1 stage, or **F**. pT2 stage, or **G**. pT3+4 stages. Forest plot based on MUC5AC expression level was used to assess the hazard ration for OS **D**. and RFS **H**. in different pT stage subgroups. *P* value was analyzed by log-rank test.

To evaluate postoperative risk of ccRCC patients with different TNM stages, subgroup analysis was performed to assess the prognostic value of MUC5AC expression. As shown in Figure 2, ccRCC patients with high MUC5AC expression had a poorer OS comparing with those with a low MUC5AC expression in the overall cohort of patients with TNM stage III + IV (hazard ratio (HR), 7.620; 95% confidence interval (CI), 4.112 – 14.120; *P* < 0.001; Figure 2C). Similarly, more adverse RFS were observed in high MUC5AC group of ccRCC patients with TNM stage III + IV (HR, 9.504; 95% CI, 4.275 – 21.127; *P* < 0.001; Figure 2G). But, no significant difference was found between high MUC5AC and low MUC5AC expression group for OS or RFS of ccRCC patients with TNM stage I (Figure 2A or 2E) and II (Figure 2B or 2F). Therefore, the ccRCC patients with TNM stage III + IV could be stratified to assess the criticality of OS and RFS according to MUC5AC expression.

**Figure 3:**
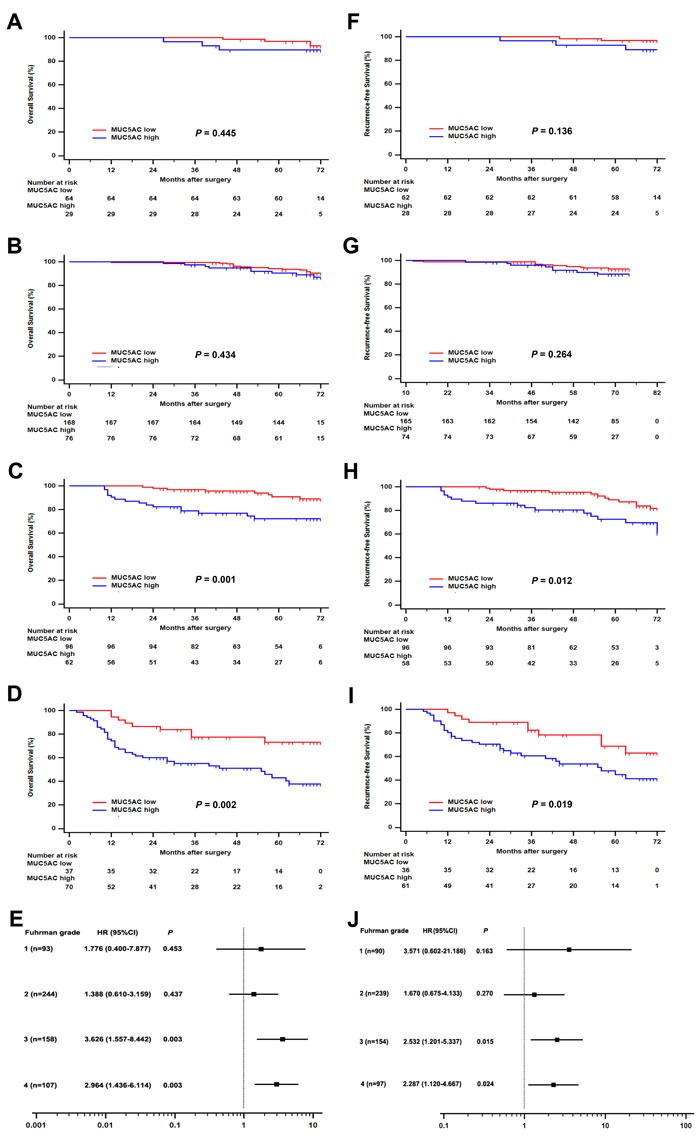
Subgroup analysis of MUC5AC prognostic value for ccRCC patients with different Fuhrman grades Overall survival (OS) for ccRCC patients with **A**. Fuhrman grade 1, or **B**. Fuhrman grade 2, or **C**. Fuhrman grade 3, or **D**. Fuhrman grade 4. Recurrence-free survival (RFS) for ccRCC patients with **F**. Fuhrman grade 1, or **G**. Fuhrman grade 2, or **H**. Fuhrman grade 3, or **I**. Fuhrman grade 4. Forest plot based on MUC5AC expression level was established to assess the hazard ration for **E**. OS and **J**. RFS in ccRCC patients with different Fuhrman grades. *P* value was analyzed by log-rank test.

Analogously, subgroup analysis was carried out to evaluate postoperative risk of ccRCC patients with different Fuhrman stages according to MUC5AC expression. Patients with high MUC5AC expression level were confirmed to have a much more adverse OS comparing with those with a low MUC5AC expression, in the cohort of patients with whether Fuhrman stages III (HR, 3.626; 95% CI, 1.557 – 8.442; *P* = 0.003; Figure 3C) or Fuhrman stages IV (HR, 2.964; 95% CI, 1.436 – 6.114; *P* = 0.003; Figure 3D). Meanwhile, more terrible RFS was found in high MUC5AC group of patients with Fuhrman grade 3 (HR, 2.532; 95% CI, 1.201 – 5.337; *P* = 0.015; Figure 3H) or Fuhrman grade 4 (HR, 2.287; 95% CI, 1.120 – 4.667; *P* = 0.024; Figure 3H). No significant correlation was found between MUC5AC expression and OS or RFS in the cohort of patients with Fuhrman grade 1 (Figure 3A or 3F) or grade 2 (Figure 3B or 3G). Taken together, the ccRCC patients with Fuhrman grade 3 or 4 could be stratified to evaluate the prognostic risk of OS and RFS according to MUC5AC expression.

### MUC5AC was identified as a potential independent adverse prognosticator for ccRCC patients

Univariate and Multivariate Cox analyses were carried out to further investigate the possibility of MUC5AC as a potential independent prognostic predictor for ccRCC patients. As shown in Table 2, the results of Univariate Cox analysis showed that, MUC5AC expression was negatively associated with OS (*P* < 0.001) and RFS (*P* < 0.001). Noteworthy, age of patients, pT-stage, pN-stage, tumor size, metastasis, LVI, Fuhrman grade, rahbdoid differentiation, sarcomatoid features, tumor necrosis and ECOG-PS were all significantly associated with OS of ccRCC patients. Besides, Tumor size, LVI, pT-stage, rahbdoid differentiation, Fuhrman grade, sarcomatoid features, tumor necrosis and ECOG-PS were also found to be correlated with RFS of ccRCC patients.

**Table 2:** Univariate and multivariate analysis of overall survival and recurrence-free survival

Characteristic	Overall survival			Recurrence-free survival		
	Univariate analysis	Multivariate analysis		Univariate analysis	Multivariate analysis	
	P*	Hazard Ratio (95% CI)	P*	P*	Hazard Ratio (95% CI)	P*
**Mean age, years†**	**0.002**	1.023 (1.003 - 1.044)	**0.028**	0.166		
**Gender**	0.142			0.273		
**Male**						
**Female**						
**Tumor size, cm†**	**< 0.001**	1.311 (1.202 - 1.430)	**< 0.001**	< 0.001	1.351 (1.239 - 1.473)	**< 0.001**
**pT stage**	**< 0.001**		**< 0.001**	< 0.001		**< 0.001**
**T1**		Reference			Reference	
**T2**		1.478 (0.687 - 3.181)	**0.321**		2.213 (0.997 - 4.914)	0.052
**T3**		3.225 (1.913 - 5.435)	**< 0.001**		3.275 (1.881 - 5.704)	**<0.001**
**T4**		8.259 (3.099 - 22.009)	**< 0.001**		7.716 (2.874 - 20.711)	**<0.001**
**pN stage**	**< 0.001**		**< 0.001**			
**N0**		Reference				
**N1**		7.023 (3.011 - 16.380)				
**Metastasis**	**< 0.001**		**0.001**			
**M0**		Reference				
**M1**		3.443 (1.686 - 7.031)				
**Fuhrman grade**	**< 0.001**		**0.002**	**< 0.001**		**< 0.001**
**1**		Reference			Reference	
**2**		1.023 (0.435 - 2.406)	**0.959**		1.096 (0.410 - 2.929)	0.856
**3**		1.452 (0.602 - 3.500)	**0.408**		2.574 (0.976 - 6.787)	0.057
**4**		2.690 (1.098 - 6.587)	**0.031**		4.652 (1.737 - 12.460)	0.002
**LVI**	**< 0.001**		**< 0.001**	**< 0.001**		**< 0.001**
**Absent**		Reference			Reference	
**Present**		3.530 (2.229- 5.592)			2.413 (1.540 - 3.782)	
**Rahbdoid differentiation**	**< 0.001**		**0.317**	**< 0.001**		0.990
**Absent**		Reference			Reference	
**Present**		1.344 (0.755 - 2.392)			1.019 (0.511 - 1.940)	
**Sarcomatoid features**	**< 0.001**		**< 0.001**	**< 0.001**		**< 0.001**
**Absent**		Reference			Reference	
**Present**		4.397 (2.229 - 8.672)			3.884 (1.849 - 8.157)	
**Tumor necrosis**	**< 0.001**		**0.002**	**< 0.001**		**< 0.001**
**Absent**		Reference			Reference	
**Present**		2.164 (1.347 - 3.476)			2.318 (1.456 - 3.693)	
**ECOG-PS**	**< 0.001**		**0.344**	**< 0.001**		**0.934**
**0**		Reference			Reference	
**≥1**		1.303 (0.755 - 2.249)			1.024 (0.590 - 1.776)	
**MUC5AC**	**< 0.001**		**0.041**	**< 0.001**		**0.004**
**Low**		Reference			Reference	
**High**		1.609 (1.023 - 2.531)			1.948 (1.243 - 3.052)	

MUC5AC, mucin 5AC; ccRCC, clear-cell renal cell carcinoma; LVI, lymphovascular invasion; ECOG-PS, Eastern Cooperative Oncology Group performance status; SD, standard deviation. *P < 0.05 is considered statistically significant.

Subsequently, all these significant risk parameters above were brought into the Multivariate Cox analysis of OS and RFS, respectively. MUC5AC expression was proved to be an independent adverse prognostic factor for OS (HR, 1.609; 95% CI, 1.023 – 2.531; *P* = 0.041) and RFS (HR, 1.948; 95% CI, 1.243 - 3.052, *P* = 0.004). Besides, tumor size, Fuhrman grade, pT-stage, LVI, ECOG-PS, sarcomatoid features, and tumor necrosis were also confirmed to be independent adverse prognostic factors for OS and RFS of ccRCC patients.

### Prognostic nomogram for ccRCC

**Figure 4:**
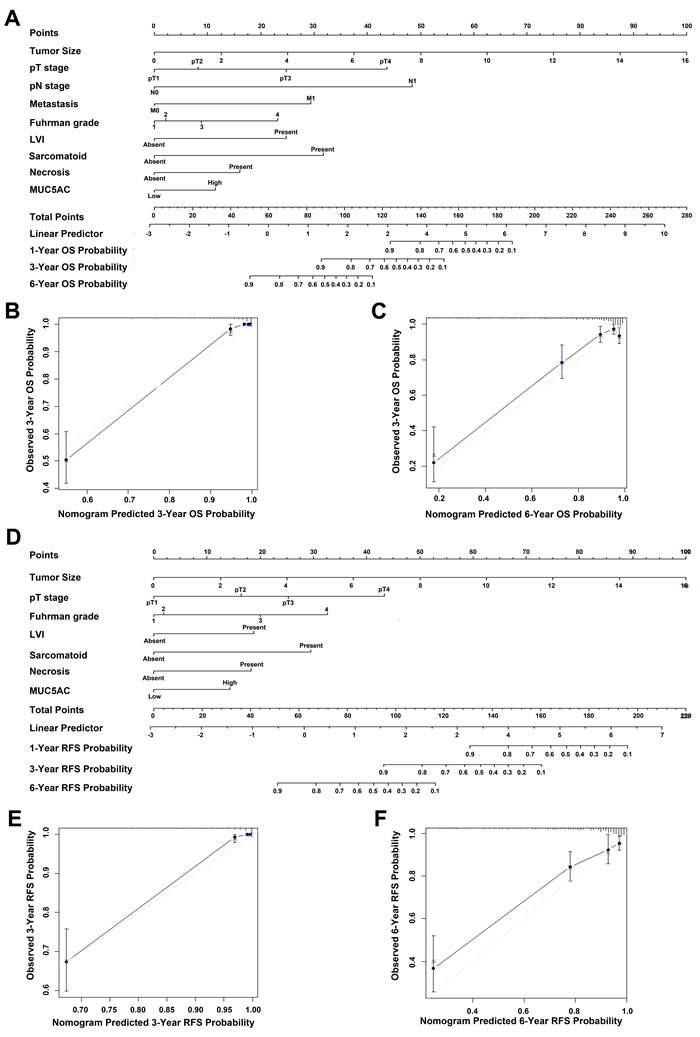
Nomogram for predicting clinical outcomes of ccRCC patients **A**., nomogram for predicting overall survival (OS) rate integrated with tumor size (continuous variable), pT-stage (pT1-pT2-pT3-pT4), metastasis (N0/N1), pN-stage (N0/N1), Fuhrman grade (1–2–3–4), LVI (absent/present), necrosis (absent/present), MUC5AC expression(low/high), and sarcomatoid (absent/present); **B**., nomogram predicted 3-year OS probability; **C**., nomogram predicted 6-year OS probability. **D**., nomogram for predicting recurrence-free survival (RFS) rate integrated with tumor size (continuous variable), Fuhrman grade (1–2–3–4), LVI (absent/present), pT-stage (pT1-pT2-pT3-pT4), sarcomatoid (absent/present), necrosis (absent/present), and MUC5AC expression (low/high); **E**., nomogram predicted 3-year RFS probability; **F**., nomogram predicted 6-year RFS probability. Vertical bars: 95% confident interval, grey line: ideal model. Higher total point suggested the probability of a more adverse clinical outcome.

A prognostic nomogram via integrating above conventional independent prognostic indicators including MUC5AC was established to predict the OS and RFS of ccRCC patients. As shown in Figure 4A, total points were demonstrated to assess the criticality of various risk factors. The higher point was, the poorer the OS of ccRCC patients might be. The calibration plot for the probability of OS at 3- or 6-year after surgery presented an optimal agreement between actual observation and the prediction by nomogram (Figure 4B and 4C), predictive accuracy of 3-year OS probability and 6-year OS probability were 93.08% and 88.51%, respectively. Similarly, the prognostic nomogram for RFS was show in Figure 5D, predictive accuracy of 3-year RFS probability and 6-year RFS probability were 96.36% and 87.32%, respectively (Figure 4E and 4F).

### Superior performance of prognostic nomogram model including MUC5AC for ccRCC

The prognostic nomogram via integrating above conventional independent prognostic indicators including MUC5AC was named as “MUC5AC-based model”. To evaluate whether MUC5AC-based model improve predictive accuracy, the C-index (Harrell's concordance index) and AIC (Akaike information criterion) of MUC5AC-based model, as well as TNM, UISS (University of California Los Angeles Integrated Staging System), SSIGN (the Mayo clinic stage, size, grade, and necrosis score), Leibovich were performed for OS and RFS analysis, respectively. As shown in Table 3, as for OS of ccRCC patients, the C-index of TNM, UISS, SSIGN and Leibovich was 0.733, 0.758, 0.798 and 0.809, respectively. Meanwhile, the C-index of MUC5AC-based model was improved to 0.885. The AIC of TNM, UISS, SSIGN and Leibovich was 1189.94, 1157.87, 1100.80 and 1111.57, respectively. The AIC of MUC5AC-based model was reduced to 988.65. As for RFS, the C-index of MUC5AC-based model was improved to 0.873, and its AIC was reduced to 918.25. These lines of evidences implicate a better predictive accuracy of MUC5AC-based model for OS and RFS of ccRCC patients.

**Table 3:** Comparison of the prognostic accuracy of the prognostic models

Model	Overall survival			Recurrence-free survival		
	C-index		AIC	C-index		AIC
MUC5AC-based model	0.885	988.65			918.25	
TNM	0.733	1189.94			1098.09	
UISS	0.758	1157.87			1055.71	
SSIGN	0.798	1100.80			1003.63	
Leibovich	0.809	1111.57			988.77	

MUC5AC, mucin 5AC; C-index, Harrell's concordance index; AIC, Akaike information criterion; TNM,; UISS, University of California Integrated Staging System; SSIGN, the Mayo Clinic stage, size, grade, and necrosis.

## DISCUSSION

In this study, MUC5AC expression was firstly found to be up-regulated in patients with ccRCC, positively associated with tumor size, pN stage, Fuhrman grade, LVI, rahbdoid differentiation, sarcomatoid features, tumor necrosis, ECOG-PS and recurrence. Furthermore, MUC5AC expression might be contributed to risk stratification of ccRCC patients with TNM stage III+IV or Fuhrman grade 3 or 4 for OS and RFS analysis, MUC5AC expression was analyzed to be negatively correlated with OS and RFS of patients with ccRCC. What's more, MUC5AC was authenticated as a potential independent adverse prognostic factor, prediction accuracy of MUC5AC-based new nomogram model was drastically improved for prognosis of ccRCC patients, compared with UISS, SSIGN and Leibovich models.

MUC5AC is a major constituent of the protective layer over the surface of respiratory tract, gastric mucosa and so on. The alteration of MUC5AC expression was found to be involved in chronic inflammation and malignant transformation in several diseases. Extracellular ATP was induced by dsRNA and released via pannexin channel in chronic obstructive pulmonary disease (COPD), which resulted in the release of MUC5AC in an autocrine manner, mainly via P2Y2 receptor [16]. Increased MUC5AC is mediated by CREB and NF-κB under the stimulus of IL-1β [17], prostaglandin F2α [18], but negatively regulated by IL-1 receptor antagonist via PLCβ3 during airway inflammation [19]. Besides, the expression of MUC5AC was up-regulated by IL-17A in nasal polyps via the Act1-mediated pathway [20], and neutrophil elastase in respiratory epithelial cells [21], and negatively regulated by Src homology 2-containing protein tyrosine phosphatase-2 via the down-regulation of the ERK1/2 MAPK pathway in the airway [22]. MUC5AC stimulated by conjugated bile acids was involved in the phosphatidylinositol 3-kinase/protein kinase C/activator protein-1 signal pathway in the esophagus [23]. Hyper production of MUC5AC in the airway epithelium was regulated by a bidirectional signal circuit between Notch and EGFR pathways in inflammatory diseases [24]. MUC5AC was found to be up-regulated in endometrial adenocarcinoma compared with normal endometrium or endometrial hyperplasia [25]. The interference of p38 gene expression was confirmed to inhibit the release of IL-1β and TNF-α in the formation and progression of hepatolithiasis, which resulted in down-regulation of MUC5AC expression [26]. MUC5AC was proved to interact with integrin β4 that mediates phosphorylation of FAK at Y397, resulting in lung cancer migration [27]. Notch3 and Jagged2 were found to contribute to the development and glandular differentiation of gastric cancer, together with MUC5AC [28]. Therefore, modulation of these MUC5AC-associated pathways may suggest a promising strategy for inflammatory and malignant diseases. In this study, the expression of MUC5AC was significantly higher in ccRCC tissues compared with the corresponding tissue adjacent to carcinoma, suggesting that MUC5AC may be involved in the development and progression of ccRCC (Figure 1A). Besides, Increased MUC5AC was found to be positively associated with tumor size, pN stage, Fuhrman grade, LVI, rahbdoid differentiation, sarcomatoid features, tumor necrosis, ECOG-PS and recurrence, which might be conducive to better understand the pathogenesis of ccRCC, even found a way to targeted therapy (Table 1). Of course, we still need a great deal of researches to further clarify the pathogenesis of kidney cancer.

The change of MUC5AC expression was demonstrated to be associated with poor prognosis of malignant tumors. MUC5AC was used as a marker of gastric phenotype in stomach tumors, and its expression was associated with tumor stage. Decreased Muc5AC expression was associated with poor prognosis in gastric cancer, especially advanced gastric cancers [11, 29]. MUC5AC hypomethylation was an independently predictor of microsatellite instability associated with colorectal cancer [30]. High expression of MUC5AC was associated with favorable outcome in colorectal cancer, notably in intermediate stages II and III [31], and absence of MUC5AC expression might be a prognostic factor for more aggressive colorectal carcinoma [32]. Increased MUC5AC were specific makers for non-terminal respiratory unit adenocarcinoma, including both mucinous adenocarcinoma and central type adenocarcinoma [33]. MUC5AC expression was a benefit to better survival of patients with pancreas invasive ductal carcinoma [34, 35]. The expression of serum MUC5AC was up-regulated in biliary tract carcinoma patients compared with that in patients with benign biliary disease or healthy control subjects [36]. In this study, high MUC5AC expression was firstly found to be negatively correlated with OS and RFS of patients with ccRCC (Figure 1B and 1C). Moreover, MUC5AC expression might be contributed to risk stratification of ccRCC patients with TNM stage III+IV or Fuhrman grade 3 or 4 for OS and RFS analysis (Figure 2 and 3). As a matter of fact, all these results suggested an important clinical significance for predicting outcomes of ccRCC patients and evaluating prognosis of postoperative ccRCC patients.

In the current clinical practice, several prognostic algorithms were established to predict outcomes of patients with ccRCC. For example, University of California Intergrated Staging System (UISS), which was set up via integrating TNM stage, Fuhrman grade, and ECOG-PS [37], SSIGN [38], and Leibovich model [39]. However, the underlining mechanisms and biological characteristics of ccRCC are complicated before we totally conquer it. The cancerous patients with the same pathological type or the comparable stage may show a great variation in tumor progression and clinical outcomes [40]. Therefore, it's urgent to establish an accurate and effective prognostic algorithm for clinical outcomes of ccRCC patients. In this research, MUC5AC was identified as an independent prognostic marker (Table 2), and incorporated into a newly-established nomogram with other independent prognostic parameters for ccRCC patients (Figure 4).

The predictive accuracy of MUC5AC-based nomogram was dramatically improved for OS and RFS of ccRCC, compared with that of UISS, SSIGN and Leibovich models, which suggested a profound clinical significance.

Although the important clinical value of MUC5AC for ccRCC patients has been demonstrated above, there are still some limitations about this research to be discussion. To begin with, a series of basic experiments should be designed and performed so as to further explore the potential molecular mechanisms of MUC5AC in the occurrence and development of ccRCC in our future work, although immunohistochemical staining of MUC5AC was carried out in this clinical research. After all, IHC analysis is always somewhat subjective. Besides, a multicenter, large sample clinical trial is essential to confirm the independent poor prognostic value of MUC5AC and predictive accuracy of MUC5AC-based nomogram.

Taken together, MUCC5AC was found to take part in the occurrence and progression of ccRCC. Increased MUC5AC was demonstrated to be conducive to risk stratification, and identified as an independent poor prognostic factor for ccRCC patients. MUC5AC-based nomogram was confirmed to remarkably improve the predictive accuracy for the prognosis of ccRCC patients. MUCC5AC could be developed as a promising therapeutic target and potential prognostic factor.
